# Spatiotemporal beam self-cleaning for high-resolution nonlinear fluorescence imaging with multimode fiber

**DOI:** 10.1038/s41598-021-96753-2

**Published:** 2021-09-14

**Authors:** Nawell Ould Moussa, Tigran Mansuryan, Charles-Henri Hage, Marc Fabert, Katarzyna Krupa, Alessandro Tonello, Mario Ferraro, Luca Leggio, Mario Zitelli, Fabio Mangini, Alioune Niang, Guy Millot, Massimiliano Papi, Stefan Wabnitz, Vincent Couderc

**Affiliations:** 1 Université de Limoges, XLIM, UMR CNRS 7252, 123 Avenue A. Thomas, 87060 Limoges, France; 2 Institute of Physical Chemistry, Polish Academy of Sciences, ul. Kasprzaka 44/52, 01-224 Warsaw, Poland; 3 DIET, Sapienza University of Rome, Via Eudossiana 18, 00184 Rome, Italy; 4 Dipartimento di Ingegneria dell’Informazione, Università di Brescia, via Branze 38, 25123 Brescia, Italy; 5 Université de Bourgogne Franche-Comté, ICB, UMR CNRS 6303, 9 Avenue A. Savary, 21078 Dijon, France; 6 Institut Universitaire de France (IUF), 1 rue Descartes, 75005 Paris, France; 7 Dipartimento di Neuroscienze, Università Cattolica del Sacro Cuore, 00168 Rome, Italy; 8 ALPhANOV, Institut d′Optique d′Aquitaine, Rue François Mitterand, 33400 Talence, France

**Keywords:** Optics and photonics, Physics

## Abstract

Beam self-cleaning (BSC) in graded-index (GRIN) multimode fibers (MMFs) has been recently reported by different research groups. Driven by the interplay between Kerr effect and beam self-imaging, BSC counteracts random mode coupling, and forces laser beams to recover a quasi-single mode profile at the output of GRIN fibers. Here we show that the associated self-induced spatiotemporal reshaping allows for improving the performances of nonlinear fluorescence (NF) microscopy and endoscopy using multimode optical fibers. We experimentally demonstrate that the beam brightness increase, induced by self-cleaning, enables two and three-photon imaging of biological samples with high spatial resolution. Temporal pulse shortening accompanying spatial beam clean-up enhances the output peak power, hence the efficiency of nonlinear imaging. We also show that spatiotemporal supercontinuum (SC) generation is well-suited for large-band NF imaging in visible and infrared domains. We substantiated our findings by multiphoton fluorescence imaging in both microscopy and endoscopy configurations.

## Introduction

Besides their relevance as a test-bed for fundamental research, MMFs have recently attracted a strong attention for their technological applications. For instance, MMFs may provide a solution to the data capacity bottleneck of optical communication links via spatial-division-multiplexing^1,2^, and scale-up the output energy of fiber lasers sources^3–6^. Linear and nonlinear imaging in microscopy and endoscopy configurations^7,8^ also provide an interesting application of MMFs. Several systems have already been proposed, which all require a careful control of multimode beam propagation^9,10^. The first images were obtained in a wide-field configuration, based on holographic techniques^11–13^. More recently, holograms have been replaced by spatial light modulators. These permit to shape and control light at the fiber input, and counteract the deleterious distortions undergone by the beam, when carried by the multiple fiber modes. These methods rely on using the inverse matrix of the recorded transmission matrix of the fiber, in order to project the desired output pattern at the fiber end^14–17^. The main serious drawback of these wide-field imaging systems is that any slight fiber movement changes the random mode coupling process. In turn, this strongly modifies the fiber transmission matrix, which thus needs to be updated by performing additional measurements. Moreover, such initial learning stage is time-consuming, which leads to significant slowing down of the imaging rate. A deep neural network approach, capable of learning the input–output relationship of MMFs, has been used to reconstruct images at the fiber output^18–20^. The first learning stage can also be used to transmit other images (not used for training), which may increase the overall robustness of the imaging system. So far, both linear and nonlinear distortions caused by propagation in MMFs have been taken into account, which allowed for the use of short pulses in the formation of NF images. Although two-photon microscopy is an imaging technique largely exploited over the last 30 years^21^, it has been recently revisited, thanks to the introduction of spatial wavefront shaping. In this way it is possible to achieve high-resolution focusing and tight spatial sectioning, even when the light has passed through a dispersive scattering medium. Spatial wavefront shaping permits the pre-compensation of the disorder experienced along the propagation, so that one can optimize both the focusing point^22,23^ and the pulse duration^24,25^ of the output beam. In this way, one can combine both high spatial resolution and efficient nonlinear imaging; an additional scanning of the sample is used to obtain the images. However, the use of spatial wavefront shaping for beam optimization at the MMF output suffers from the same drawbacks as the aforementioned wide-field imaging technique. Namely, the approach requires a non-negligible time to learn, and has a strong sensitivity to the random mode coupling variations, thus weakening the performances of the imaging system.

In the recent experiment of photoacoustic endoscopy^8^, the resolution improvement, obtained by replacing a step-index with a GRIN multimode fiber, was ascribed to the presence of spatial BSC^26–34^. However, the spatial reshaping of the pump beam as a function of the pump power was not proven. By using a 100 µm core diameter GRIN optical fiber, a resolution of only 30 µm was demonstrated, which is 28 times larger than the pump wavelength. Therefore, no high-resolution performance was demonstrated, and the main issue of image stability against fiber manipulation, a key property of BSC, was not investigated.

Finally it should be mentioned that the SC-based multiphoton imaging has significant advantages and large benefits. Low-cost, compact and robust systems were reported in the recent studies^35^. Simultaneous multiband excitation is very promising for in-vivo imaging. So the new systems and approaches of such a multiband sources are still very interesting.

In order to enhance the performance of nonlinear imaging systems up to their limits, the full spatiotemporal dynamics of the self-cleaning process can be exploited. This is the purpose of this Letter: we demonstrate that the spatiotemporal character of BSC leads to a significant resolution enhancement in multispectral-multiband multiphoton fluorescence imaging, in both microscopy and endoscopy configurations. This is obtained thanks to BSC-induced energetic broadband frequency conversion, activated by significant temporal pulse narrowing, and high-energy multimode soliton generation^27–30,36–40^.

## Results

**Figure 1:**
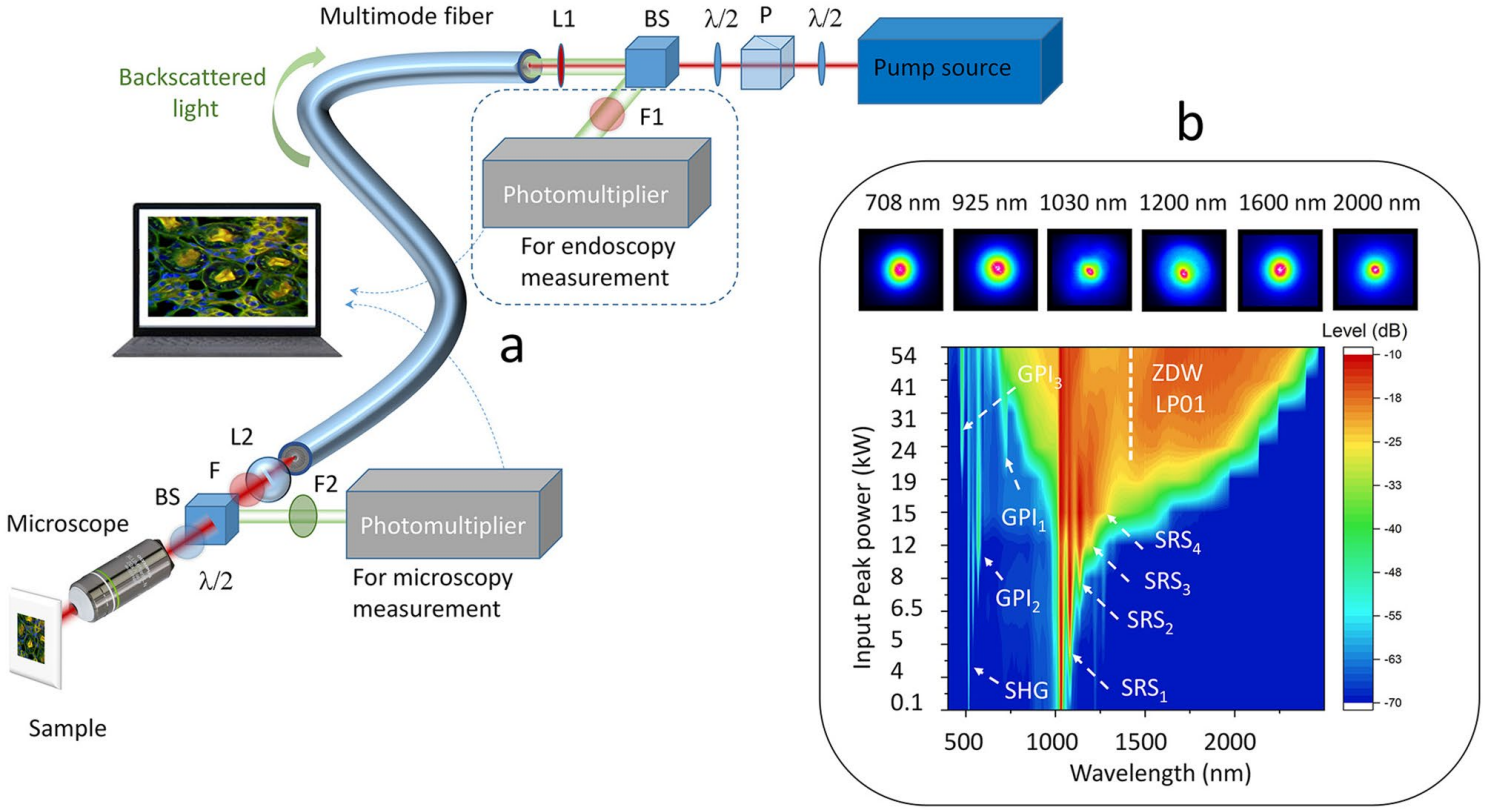
Experimental setup and characteristics of the output self-cleaned beam. (**a**) Schematic representation of the experimental setup as detailed in “Methods” section (λ/2: half-wave plate, P: polarizer, BS: beam splitter, F–F1–F2: Spectral filters, L1–L2: convergent lenses). (**b**) SC generation vs. input peak power, obtained in an 18 m long, 50/125 µm GRIN optical fiber (3 m for BSC); Beam profiles at the fiber output, for different wavelengths.

In our experiments (Fig. 1a) we coupled a Gaussian pulsed (80 ps) beam at 1064 nm into 3-m or 18-m spans of GRIN MMF, followed by a multiphoton microscope (Thorlabs Bergamo). The microscope was composed of a scanning system, a microscope objective (LUMFLN60XW, Olympus), a set of band-pass filters coupled with dichroic mirrors, and two photomultiplier tubes (see “Methods”) placed before, or after the MMF, in order to measure the emitted fluorescence in microscopy, or endoscopy configurations.

By measuring its spatiotemporal features, we first characterized the BSC effect that occurs in the GRIN MMF. The evolution of the output spatial beam profile, with respect to the input peak power, is shown in the supplementary information section (Supplementary Fig. SM1). Spatial beam self-cleaning occurs at peak pulse powers above 10 kW, leading to a typical bell-shaped beam pattern, surrounded by a low-energy speckled background (see Fig. SM1 in supplementary material). The characterization of the power proportion in this bell-shaped mode was previously evaluated in Ref.^28^, and it is directly dependent upon: (i) the initial pump peak power, (ii) the number of excited modes, (iii) the fiber length. In our present experiments, this proportion can be estimated to be close to 80%. In the temporal domain, we measured a three-fold pulse narrowing; the autocorrelation traces and the corresponding pulse durations (assuming a Gaussian pulse shape) are shown in Supplementary Fig. SM2 of the supplementary information. Thus, BSC simultaneously improves the output beam quality, and doubles the output pulse peak power (for the given fix average power). By further increasing the peak pulse power, the initially nearly monochromatic spatiotemporal beam shaping turns into a multicolor one, because of the self-phase-modulation induced spectral broadening. Figure 1b shows the SC, generated at the fiber output, extending from 0.6 µm up to 2.4 µm. As the pump pulse power is increased above the threshold for BSC, we observe first a stimulated Raman scattering (SRS) cascade up to 1.3 µm (Fig. 1b). Beyond this wavelength, which represents also the zero-dispersion wavelength (ZDW) of the LP01 mode, a part of the pulse energy propagates in the anomalous dispersion regime, which leads to soliton generation. As well-known, soliton propagation is subject to the Raman-induced soliton self-frequency shift, which leads to spectral broadening towards the 2 microns region^27–30,36–40^. Note that the generated multimode solitons feature very high (up to the MW range) peak powers, which permits to drastically improve the quality of the nonlinear imaging of biological samples via two-photon absorption (2PA) and three-photon absorption (3PA), when using infrared light. Because of multimode soliton dynamics and the associated spatiotemporal oscillations induced by self-imaging, dispersive wave generation is obtained, feeding the near-infrared spectrum between 700 and 1000 nm^37–39^. Additionally, we clearly observed geometric parametric instability (GPI) sidebands, leading to frequency conversion of the input pump into spectral anti-Stokes and Stokes sidebands with large (i.e. > 100 THz) frequency detuning in both the visible and the infrared domains. We may identify sharp spectral peaks close to 730 nm, 560 nm and 480 nm, leading to corresponding Stokes waves in the infrared and far infrared domain. These observations are in full agreement with published papers on GPI and SC generation in GRIN optical fibers^26–30^. We would like to underline that the output beam pattern was found to remain highly robust against environmental perturbations, e.g., fiber bending and squeezing.

**Figure 2:**
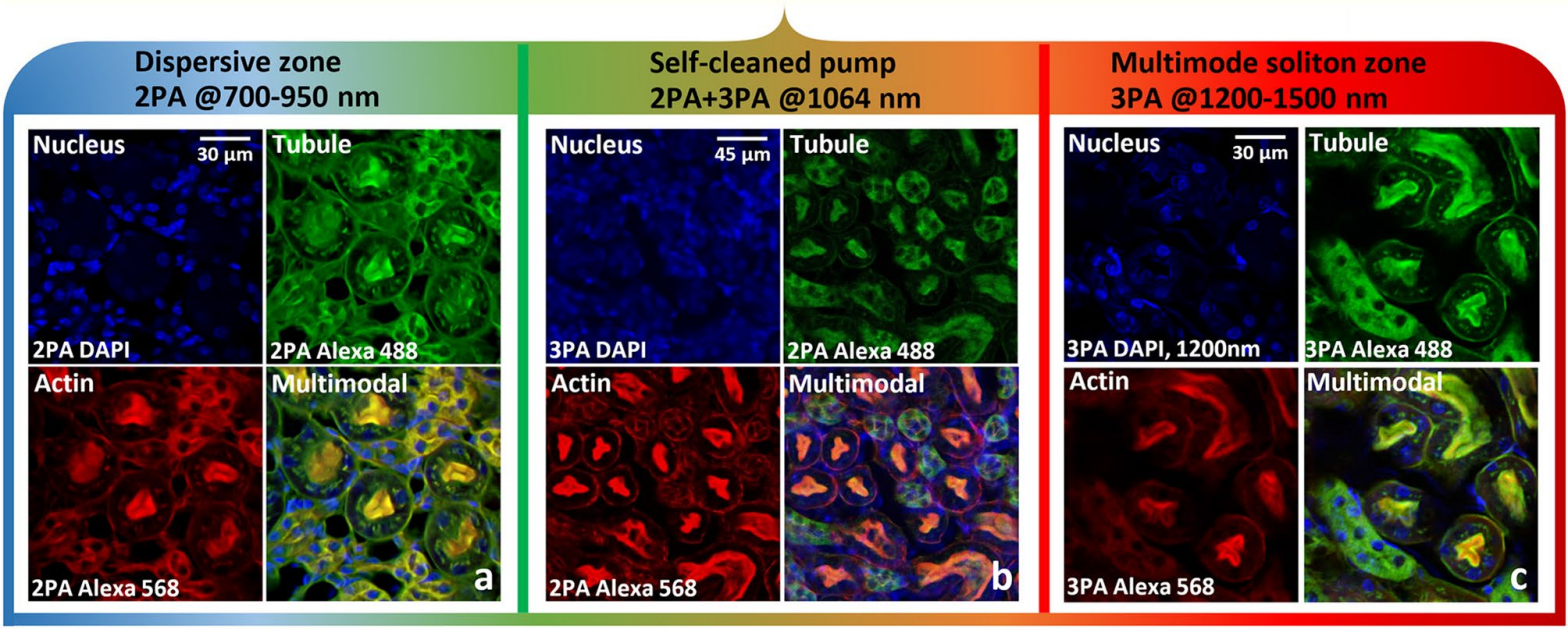
NF images of mouse kidney, labelled with Alexa 488, Alexa 568 and DAPI, revealing tubule, actin and nucleus, respectively. Microscopy configuration. (**a**) 2PA fluorescence imaging (tubule, actin and nucleus) by using visible/IR light (700–950 nm). (**b**) 2PA and 3PA (nucleus) imaging, by using a self-cleaned pump beam at 1064 nm. (**c**) 3PA imaging by using IR light (1300–1500 nm; 1200 nm for nucleus). Dwell time: 5 µs/px; averaged traces for 1 image: 20; image size: 1024 × 1024 pixels.

Next, we used the self-cleaned beam at 1064 nm (only) to perform NF imaging of kidney of mouse (Fig. 2b) and bovine endothelial cells (Supplementary Fig. SM3b). The total average power sent on the biological sample was limited to a few 5 mW with 200 kHz repetition rate. We obtained images of tubule, actin and nucleus, labelled with Alexa 488/BODIPY, Alexa 568/Texas Red and DAPI respectively. Imaging mechanism is the 3PA for DAPI, and the 2PA for the other fluorophores.

By increasing the peak power coupled in the GRIN fiber, we could generate a SC ranging from 700 nm to 2.4 µm. By using an optical filter (see “Methods”), we sliced from the SC a wavelength range in the near-infrared domain between 700 and 950 nm, and performed NF imaging. Images were obtained with 2PA fluorescence (Fig. 2a). As previously remarked, all of the images remained strongly stable with respect to fiber perturbations. This is because the SC was carried by a robust bell-shaped spatial beam across its entire wavelength range.

We performed equivalent experiments in the infrared domain, between 1300 and 1500 nm, and at the specific wavelength of 1200 nm, to excite DAPI fluorophore by three-photon absorption. Multimode solitons exist for wavelengths longer than the range of 1300–1350 nm, which contains the ZDW for the low-order modes that carry the main portion of the output power. The average power sent on the sample remained the same as in previous experiments. The obtained images (Fig. 2c) lead us to conclude that operating in the multimode soliton regime significantly improves the efficiency of the nonlinear imaging process.

**Figure 3:**
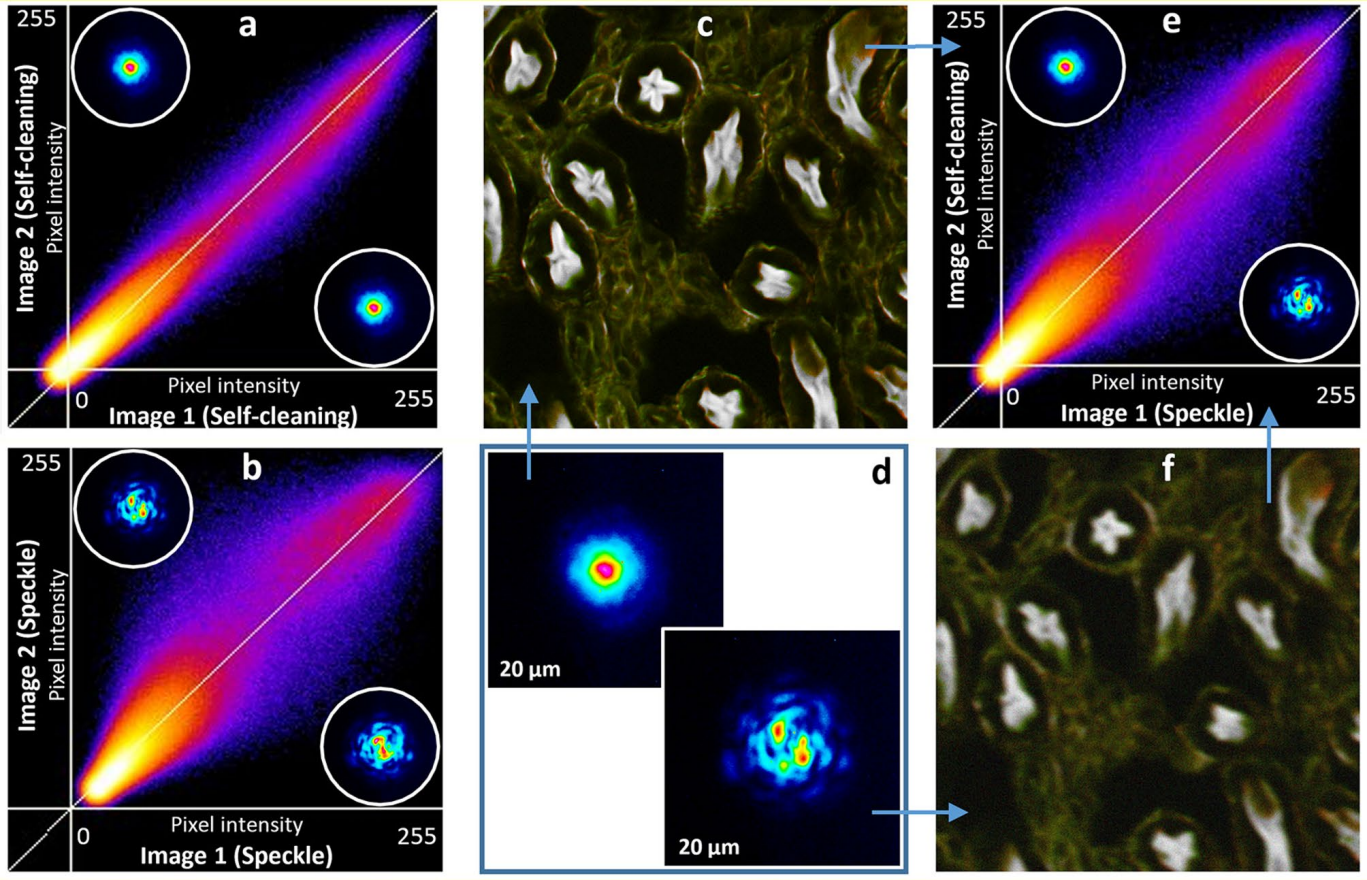
Illustration of self-cleaning robustness, resulting to the image stability. Intensity correlation (ImageJ technics- correlation of the pixel intensities) of colocalizing objects is used. Continuous squeezing is applied to the multimode fiber for (**a**) and (**b**). (**a**) Intensity correlation between two different images, recorded with the self-cleaned output beam at 1064 nm. (**b**) Same for the speckled output beam. (**d**) and insets: 2D images of the output beams with (Left) and without (Right) spatial self-cleaning. (**e**) Comparison between images recorded with a speckled and a self-cleaned beam. (**c**) Example of images, used for intensity correlation, taken with a self-cleaned output beam. (**f**) Exactly the same zone, with the speckled output beam.

In order to demonstrate the advantage of our setup, we compared the stability of NF imaging in two different situations. Specifically, when using a self-cleaned pump beam, or a speckled pump beam with the same average power, we computed (for details, see Ref.^41^) pixel intensity correlations between two images, taken with 2PA fluorescence. The biological sample was the kidney of mouse (Invitrogen FluoCells n°3 F24630), excited at 1064 nm, and analyzed at 640 nm, in order to observe actin, labelled with Alexa Fluor 568. All of the images were taken with a continuously squeezed coiled optical fiber, in order to vary the random mode coupling process inside the fiber. In the first step, by keeping certain time delay, two different images of the same sample were taken, by using the spatial self-cleaning process (Fig. 3d Left). The intensity correlation is mainly distributed along the diagonal direction (Fig. 3a), which demonstrates a strong replication of images taken from the same sample, but with a delayed time.

The estimated correlation coefficient is close to the 98.5%, calculated by ImageJ correlator. To the contrary, two different images, recorded with a speckled beam (Fig. 3d Right) at the fiber output, undergo significant distortions, as it can be clearly seen on the correlation diagram of Fig. 3b. In this case, the image correlation is also mainly distributed along the diagonal axis, but with a significant spreading, as testified by the less than 90% correlation coefficient (it can reach even lower values, depending on the strength of the externally imposed fiber squeezing).

We have also compared images taken with self-cleaned beam versus speckled beam (Fig. 3e). Result is quite predictable: self-cleaned beam significantly increasing resolution, contrast, efficiency and signal-to noise ratio of imaging (Fig. 3c,f). The speckle increases the image fuzziness.

Besides the image stability, we also investigated the impact of temporal pulse reshaping (shortening) and accompanying BSC. Both linked phenomena leads to NL fluorescence efficiency drastic gain, along with resolution improvement. DAPI fluorophore was examined to show that (Supplementary Fig. SM4). When the beam propagates in the GRIN fiber in linear regime, the signature of the DAPI fluorophore remained low, and no image of nucleus could be obtained. The same experiment was performed with a self-cleaned beam, by keeping the average power on the sample unchanged. This time, a clear signature of nucleus was obtained via 3PA fluorescence imaging, thanks to the increased pulse peak power and resolution, hence demonstrating the significant beneficial impact of BSC.

**Figure 4:**
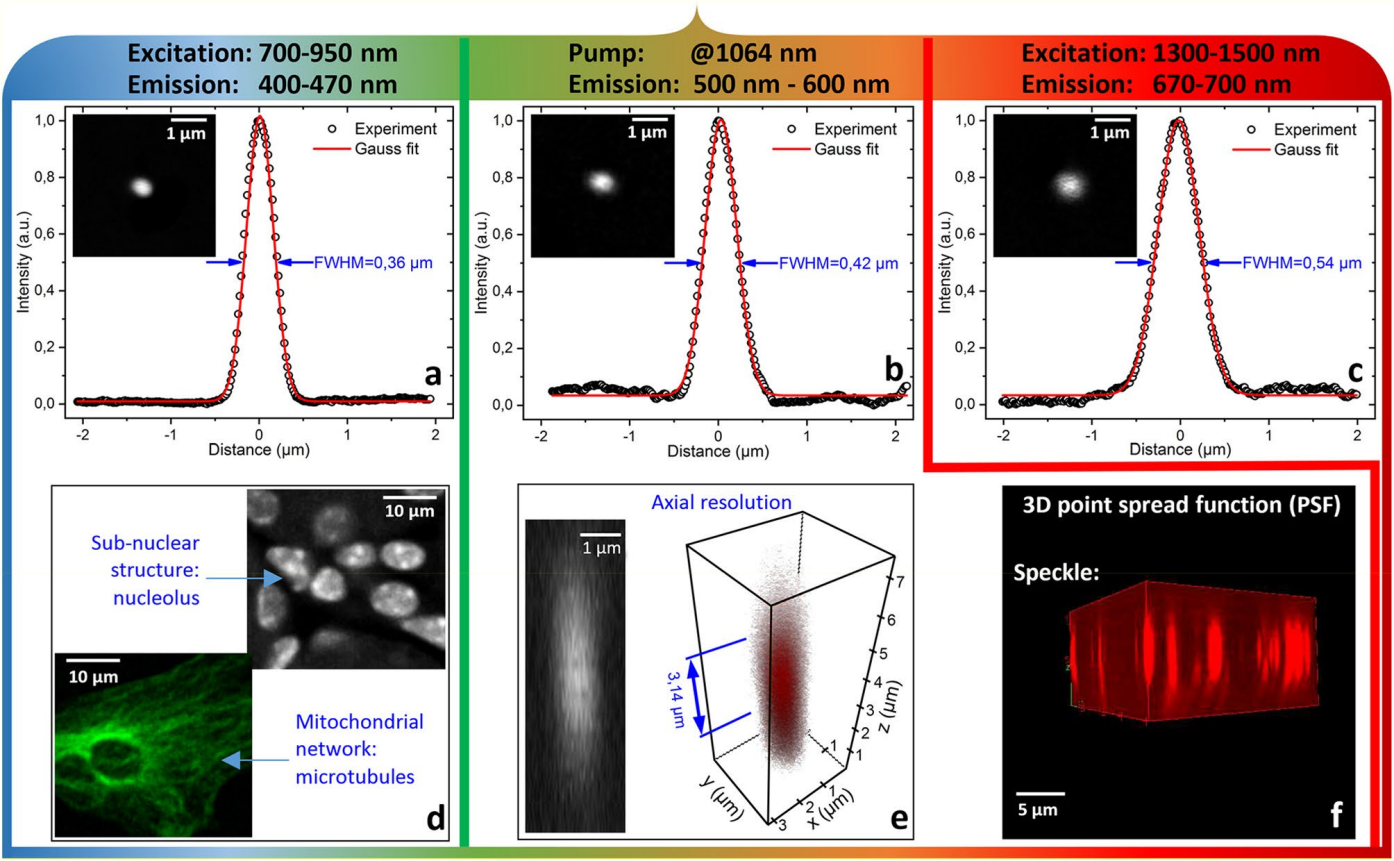
Measure of the spatial resolution of our fluorescence imaging microscopy system, based on 2PA, for three different wavelength domains. Lateral resolution in the range of 700–950 nm (**a**), 1300–1500 nm (**c**) and for 1064 nm only (**b**). (**d**) Example of high-resolution images, showing sub-nuclear structure (nucleolus) and microtubules. (**e**) Axial resolution at the pump wavelength. (**f**) 3D PSF for a speckled beam at 1064 nm.

To quantify the advantages of our new imaging system, we determined its spatial 3D and 2D resolutions. The results are shown in Fig. 4. The self-cleaned beam at 1064 nm permits transverse and longitudinal resolutions of 0.42 µm and 3.14 µm respectively (Fig. 4b,e). These results represent a significant improvement with respect to the linear multimodal regime, where the multiple focal points, caused by the initial speckled beam, greatly weaken the spatial resolution beyond 1.5 µm. The 3D PSF of speckled beam is shown on Fig. 4f. The spatial quality of the output beam is slightly improved for the generated SC, because of SRS beam clean-up and nonlinear conversion via dispersive wave generation. For near infrared (750–950 nm) and infrared (1300–1500 nm) region, by using self-cleaned beam, we can reach the maximum resolution allowed by our system, i.e. 0.36 µm and 0.54 µm respectively (Fig. 4a,c). Importantly, all studied configurations allowed to clearly resolve the sub-nuclear structure (nucleolus), and mitochondrial network (microtubules) (Fig. 4d).

**Figure 5:**
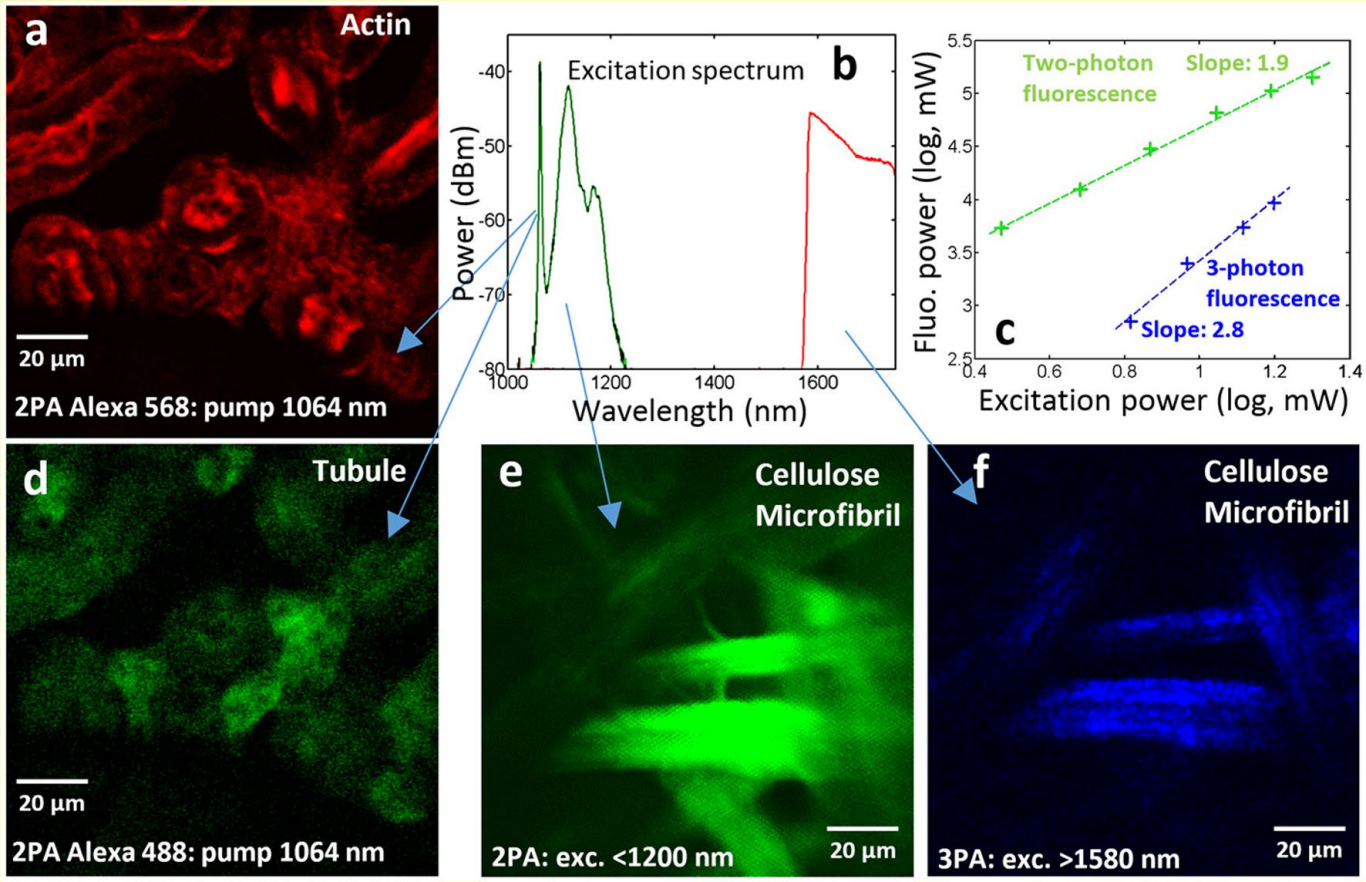
NF images, obtained in endoscopy configuration, of mouse kidney labelled with Alexa 488 and Alexa 568, revealing tubule and actin. 2PA fluorescence images of actin (**a**) and tubule (**d**) by using the self-cleaned pump beam at 1064 nm. (**e**), (**f**) 2PA and 3PA fluorescence images of cellulose microfibril. (**b**) Output spectrum used for experiments. (**c**) Fluorescence evolution versus input peak power.

Finally, in the last experiment, we studied the possibility to obtain NF images in the endoscopic configuration. First, we used the pump beam wavelength to record two-photon fluorescence of actin and tubule, labelled with Alexa 568 and Alexa 488, respectively (Fig. 5a,d). We measured the evolution, versus input peak power, of fluorescence on cellulose microfibril, in order to confirm the multiphoton nature of the nonlinear absorption process (Fig. 5c). For higher input peak powers, we generated a SC up to 2 µm. By selecting the infrared part of the SC beyond 1.55 µm (Fig. 5b), we obtained 3PA fluorescence imaging of cellulose microfibril (Fig. 5f, to be compared with 2PA imaging in Fig. 5e). When operating in the anomalous dispersion regime, multimode soliton propagation drastically increases the pulse peak power, thus facilitating high-order multiphoton nonlinear imaging. However, in contrast with the microscopy configuration, in the endoscopic configuration the image contrast of actin and tubule was reduced. This could be due to nonlinear conversion into the visible region via SC generation, which cannot be filtered out. Indeed, no filter can be placed between the fiber and the sample, in order to clean the incident beam of any visible stray light, due to the need to collect the fluorescence signal, which is detected after its backward propagation through the fiber. The use of shorter fibers could mitigate the generation of geometric parametric instabilities, and improve the signal-to-noise ratio.

## Discussion

In summary, we experimentally demonstrated that ultrafast spatiotemporal BSC based on nonlinear optical pulse propagation in a GRIN fiber can be exploited for high-resolution, multispectral and environmentally robust MMF-based NF imaging of biological samples. BSC provides high quality spatial beam bell-shape all-over the entire SC range, thus approaching the imaging resolution to its optical limits. Temporal pulse narrowing associated with BSC was found to increase the output pulse peak power, thus strongly enhancing the efficiency of multiphoton imaging. This especially occurs with a pump wavelength beyond 1.3 µm, because of the generation of high-energy multimode solitons. Spectral broadening, associated with spatiotemporal beam cleaning, can be optimized to cover the entire spectral range spanning from the visible to the infrared domain, by generating a SC carried by a cleaned spatial profile, further allowing for nonlinear imaging in a wideband configuration. The other key feature of our system is the high stability of the imaging measurement, which opens the way for the development of high resolution, environmentally-robust microscopic and endoscopic fluorescence imaging systems based on MMFs. The capability to provide both high peak and average power is another advantage of employing MMFs, which can pave the way for multispectral LIDAR applications. Our results provide the building blocks for harnessing the complexity of nonlinear spatiotemporal multimode dynamics, and developing novel bio-imaging techniques with greatly improved performances.

## Methods

### Microscopy experiments

We used a mode-locked pump laser, delivering Fourier transformed pulses (80 ps) at 1064 nm with adjustable repetition rate up to 2 MHz and a peak power up to 1 MW. The infrared linearly-polarized Gaussian beam was coupled in a 50/125 GRIN MMF by using 50 mm converging lens. Its core diameter was 52.1 µm, with a numerical aperture of 0.205. The focused input beam had a 30 µm diameter at full width at half the maximum intensity (FWHMI). Thus, 99% of the guided input power was coupled into about 80 modes. The angle injection was properly adjusted to be 0°, to favor self-cleaning process on the fundamental mode. We also used a polarizer cube, placed in between two half-wave plates, in order to control the beam power while adjusting the input beam polarization orientation. With a 3 m long fiber span, we obtained BSC without any significant spectral broadening^4^. In a second step, by increasing both the fiber length (18 m) and the input peak power (up to 50 kW), spectral broadening of the input pump pulse was obtained, until a SC was generated, covering the full wavelength range between 600 nm and 2.5 µm. At the fiber output we placed an elliptical reflective lens of 15 mm focal length and a half-wave plate; to collimate the output beam and to adjust its polarization orientation. An adjustable neutral density filter was placed at the input of microscope, in order to control the power sent on samples. Three different filters, coupled with the appropriate control of the input peak power, were used to select the desired wavelength bands: a bandpass filter FL-1064-10 (Thorlabs), a longpass filter FELH1300 (Thorlabs) and a shortpass filter FESH950 (Thorlabs), were used to select the spectral content of the pump beam at 1064 nm, 700–950 nm and 1300–1500 nm ranges, respectively. A longpass filter FELH1200 (Thorlabs) was used to select wavelengths close to 1200 nm, in order to excite the nucleus labelled with DAPI. Next, the filtered beam was sent in an upright multiphoton microscope (Bergamo-Thorlabs) with galvanometric scanners and two photomultipliers. In order to focus the incident beam on the sample, we used two microscope objectives (Olympus, XLUMPLFLN20XW and LUMFLN60XW). Three additional single-band bandpass filters at 460/80 nm, 525/50 nm and 607/70 nm from Semrock (Brightline) were inserted in front of the photomultipliers, in order to select the corresponding fluorescence emission. An afocal system composed of two converging lenses was introduced after the GRIN MMF, in order to obtain a spatial magnification of the beam, thus allowing for covering of the entire input window of the objective, and reaching the maximum spatial resolution. Images with 1024 × 1024 pixels were recorded with a dwell time of 5 µs/px with twenty accumulations. The reference of the beads used to measure the spatial resolution is: “TetraSpeck™ Fluorescent Microspheres-T14792”.

### Endoscopy experiments

The laser source used for the endoscopy experiments was the same as that used in the microscopy configuration. BSC was also obtained in the same conditions. An additional longpass filter at 700 nm was placed at 45° before the input GRIN fiber, in order to reflect the backward fluorescence generated in the samples towards two additional photomultipliers. A shortpass filter at 750 nm (FES750, Thorlabs) and a longpass filter at 450 nm (FEL450, Thorlabs) were used to select the corresponding fluorescence band in the front of the photomultipliers.

### Biological samples for microscopy experiments

We used multilabeled cell preparations from mouse kidney section provided by Invitrogen (FluoCells n°3 F24630), and labelled with Alexa 488, Alexa 568 and DAPI. A second sample was bovine pulmonary artery endothelial cells (FluoCells n°2 F14781) labelled with DAPI, BODIPY FL and Texas Red (the corresponding results are displayed in the Supplementary Information). Two additional samples of cellulose microfibril were used for the endoscopy experiments.

## Supplementary Information

Supplementary Information.
