# Supplementary material for: Spatiotemporal beam self-cleaning for high-resolution nonlinear fluorescence imaging with multimode fiber
Source: Sci Rep. 2021 Sep 14;11:18240. doi: 10.1038/s41598-021-96753-2 (PMC8440782; doi:10.1038/s41598-021-96753-2)
Supplement: Supplementary file 1 — Supplementary Information. [file 41598_2021_96753_MOESM1_ESM.docx]

Supplementary information for **“Spatiotemporal beam self-cleaning for high-resolution nonlinear fluorescence imaging with multimode fiber”**

**Nawell Ould Moussa^1^, Tigran Mansuryan^1^, Charles Henri Hage^1^, Marc Fabert^1^, Katarzyna Krupa^2^, Alessandro Tonello^1^, Mario Ferraro^3^, Luca Leggio^3^, Mario Zitelli^3^, Fabio Mangini^4^, Alioune Niang^4^, Guy Millot^5,6^, Massimiliano Papi^7^, Stefan Wabnitz^3^, and Vincent Couderc^1,*^**

*^1^ Université de Limoges, XLIM, UMR CNRS 7252, 123 Avenue A. Thomas, 87060 Limoges, France*

*^2^ Institute of Physical Chemistry, Polish Academy of Sciences, ul. Kasprzaka 44/52, 01-224 Warsaw, Poland*

*^3^ DIET, Sapienza University of Rome Via Eudossiana 18, 00184 Rome, Italy*

*^4^ Dipartimento di Ingegneria dell’Informazione, Università di Brescia, via Branze 38, 25123, Brescia, Italy*

*^5^ Université de Bourgogne Franche-Comté, ICB, UMR CNRS 6303, 9 Avenue A. Savary, 21078 Dijon, France*

*^6^ Institut Universitaire de France (IUF), 1 rue Descartes, 75005 Paris, France*

*^7^ Dipartimento di Neuroscienze, Università Cattolica del Sacro Cuore, 00168 Rome, Italy*

e-mail* [Vincent.couderc@xlim.fr](mailto:Vincent.couderc@xlim.fr)

**2D spatial evolution of the output beam versus input peak power**

Spatiotemporal beam reshaping by using Kerr-induced beam self-cleaning is based on four-wave mixing processes between all the excited transverse modes of the fiber. This parametric effect is phase-matched by the periodic modulation of the refractive index, which is obtained by mixing the self-imaging effect with Kerr nonlinearity. Thus, orthogonality between modes is broken, and a transfer of energy between them can be obtained. This progressively reshapes, as the input power grows up, the initially speckled (owing to random mode coupling) output beam. Such spatial self-cleaning eventually leads to a robust final output beam state, exhibiting an energetic fundamental mode, sitting on a remaining speckled background. The evolution of the spatial beam output pattern is shown on **Fig. SM1**.


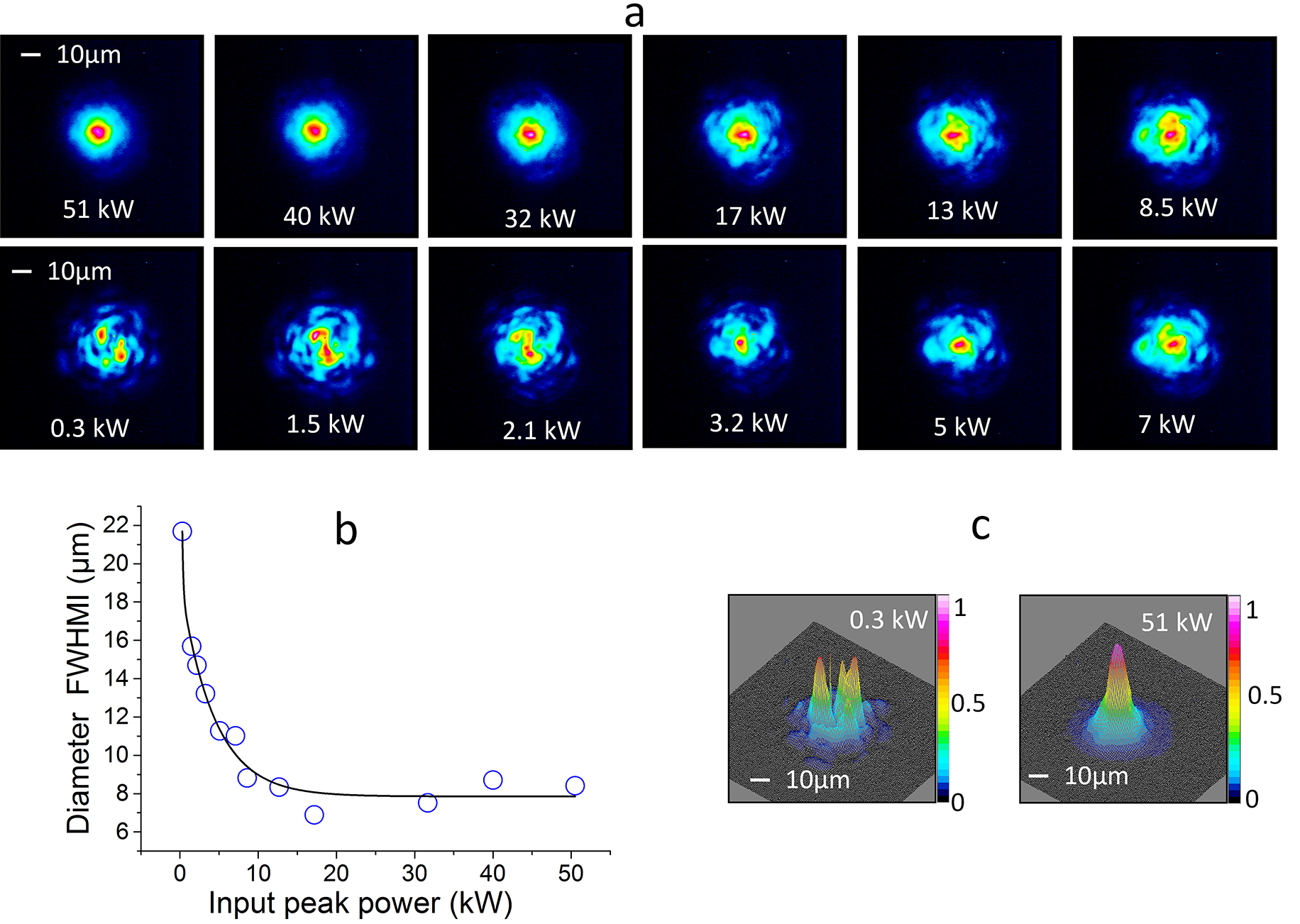


**Figure SM1.** Spatial output pattern evolution of the beam, versus input peak power. (**a**) 2D representation. (**b**) Output beam diameter FWHMI (Full width at half maximum of intensity) versus input power. (**c**) 3D representation of the output beam profile at minimum and maximum peak powers. The 50/125 GRIN MMF length, laser wavelength, pulse duration and repetition rate are: 3 m, 1064 nm, 80 ps and 200 kHz, respectively.

In our case, the self-cleaning threshold is estimated to be close to 10 kW when a fiber length close to 3 m is employed. We used a 50/125 graded-index multimode fiber (GRIN MMF). For a low input peak power, the output pattern is significantly intensity modulated, with several spots constituting the output speckled beam. By increasing the input peak power, the energy carried by high-order modes is relocated towards the fundamental mode, which significantly changes the beam envelope, exhibiting a dominant bright spot in the center (**Fig. SM1a**). Such phenomenon manifests at relatively low input peak powers, before any significant self-phase modulation occurs [1, 2]. Because of the residual energy carried in the speckled background (in our case this is close to 20%), the output beam is not completely cleaned. However, the central spot in the output beam exhibits a bell-shape profile, with a beam diameter close to that of the fundamental mode of the fiber (**Fig. SM1b** and **SM1c**).

**Temporal evolution of the output beam at 1064 nm versus input peak power**

For a low input peak power, the autocorrelation trace of the pump pulse exhibits a Gaussian shape with a duration of 120 ps, which means 80 ps pulse duration, when assuming a pure Gaussian pulse profile. By increasing the peak power and because of the temporal self-cleaning process [3], the pulse undergoes a temporal narrowing down to 26 ps (Autocorrelation: 38 ps (FWHMI)), as we can see on **Fig. SM2a** and **SM2b**.


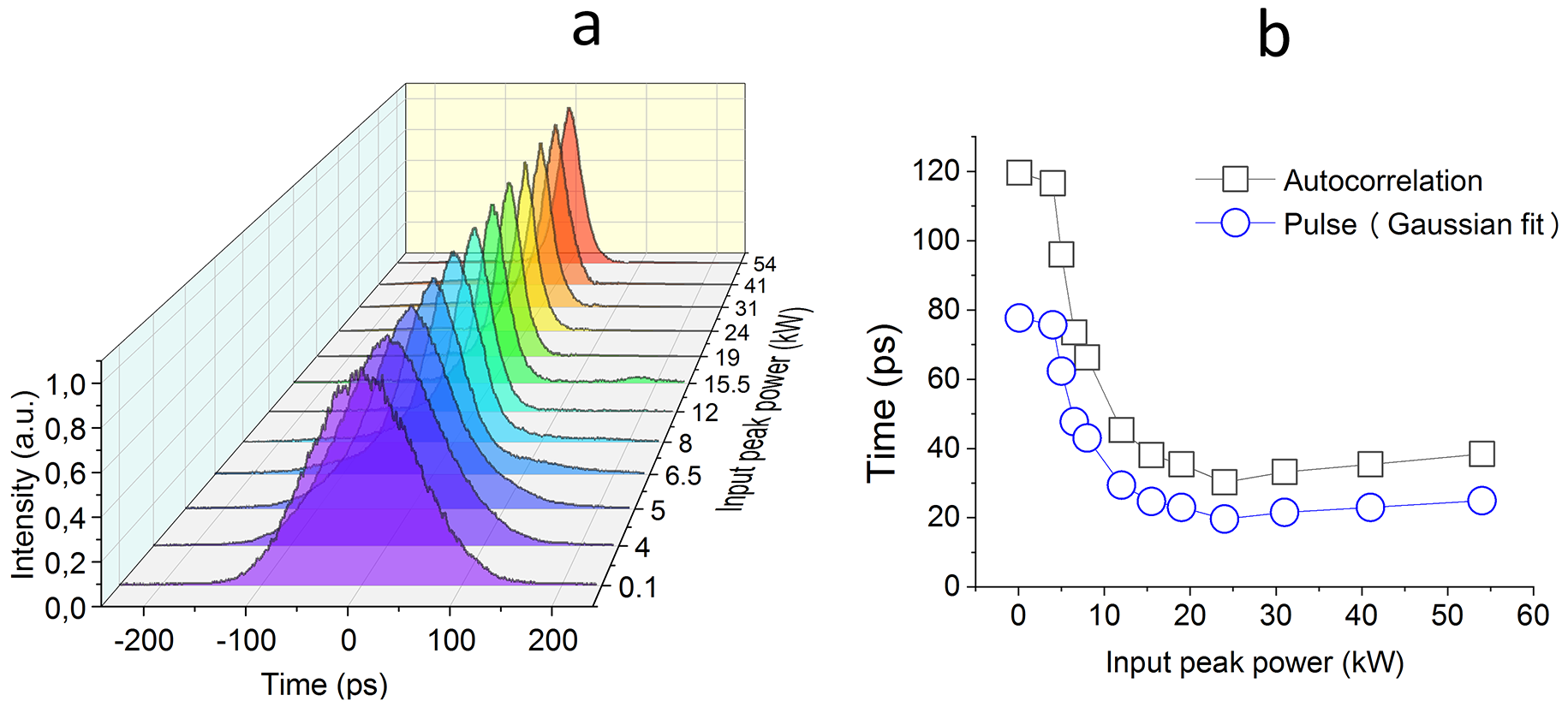


**Figure SM2.** Pump pulse temporal evolution versus input peak power. (**a**) Autocorrelation profiles. (**b**) Autocorrelation and pulse duration (FWHMI). The 50/125 graded-index multimode fiber length, laser wavelength, pulse duration and repetition rate are: 3 m, 1064 nm, 80 ps and 200 kHz respectively.

**Images of bovine endothelial cells**

Beyond the images of kidney of mouse, we realized the same experiment with bovine endothelial cells. DAPI, BODIPY FL and Texas Red are used for labelling nucleus, microtubules and actin, respectively (**Fig. SM3**). The use of pump wavelength at 1064 nm allows us to obtain two and three-photon fluorescence images that reveal microtubules, actin and nucleus, respectively (**Fig. SM3b**).

For the wavelengths lower than the pump, only two-photon imaging is possible (**Fig. SM3a**). Indeed, the nonlinear conversion process feeding the supercontinuum is mainly provided by dispersive wave generation. In this case, the peak power of frequency-converted waves is significantly lower than that obtained in the anomalous dispersion regime, where multimode solitons are generated. To the contrary, for wavelengths between 1300 nm and 1500 nm, the multimode soliton sea, owing to the fission and subsequent Raman self-frequency shift of the input high-order soliton pulse, can significantly increase the peak power inside the MMF. Thus, three-photon fluorescence images can be obtained in this case for all of the three fluorophores (**Fig. SM3c**). Only the DAPI fluorophore, exited at 1200 nm, does not benefit from solitonic propagation, but only from the temporal shortening introduced by the self-cleaning process. The stability of obtained images is again robust versus fiber bending and squeezing.


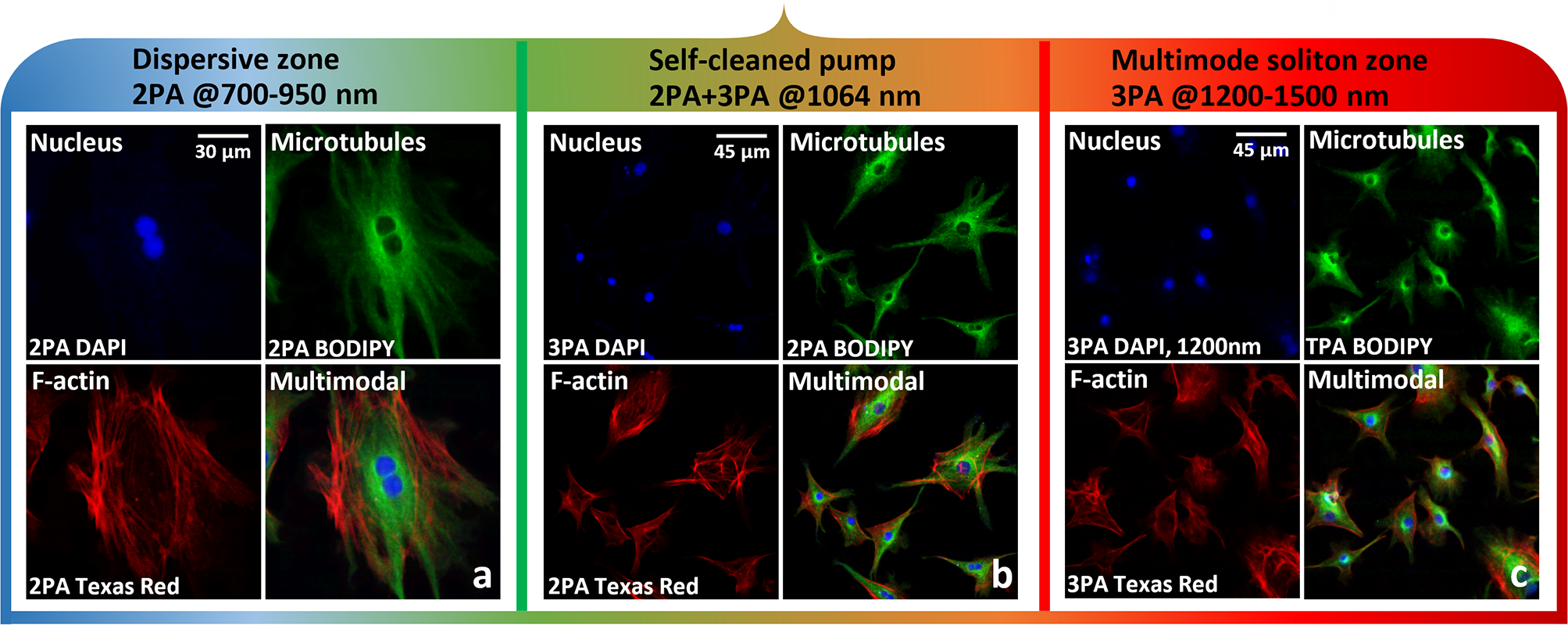


**Figure SM3.** Nonlinear fluorescence images, obtained in microscopy configuration, of bovine endothelial cells, labelled with DAPI, BODIPY FL and Texas red. (**a**) Two-photon fluorescence images of tubule, actin and nucleus with wavelengths between 700 and 950 nm. (**b**) Three-photon fluorescence images of nucleus, and two-photon fluorescence images of actin and microtubules by using a self-cleaned pump beam at1064 nm. (**c**) Three-photon fluorescence images of tubule, actin and nucleus, by using infrared light between 1300 nm-1500 nm and only 1200 nm for DAPI. Dwell time: 5µs/pixel, averaged traces for 1 image: 20, image size: 1024x1024 pixels.

**Temporal pulse peak power increase, accompanying the spatial beam self-cleaning process: application to three photon experiments on nucleus labeled with DAPI**

Beyond the improvement of beam brightness resulting from spatial self-cleaning, temporal pulse reshaping, which accompanies spatial beam cleanup, leads to an increase of the output pulse peak power [3]. Autocorrelation traces of the output pump beam, versus the input peak power, are displayed on the **Fig. SM4c**. In our experimental conditions, we observed three-fold temporal pulse shortening. Namely the output pulse duration was reduced from the initial pulse value of 82ps (autocorrelation trace of 119 ps) down to the value of 27 ps (autocorrelation trace of 39 ps), by supposing a Gaussian pulse profile.


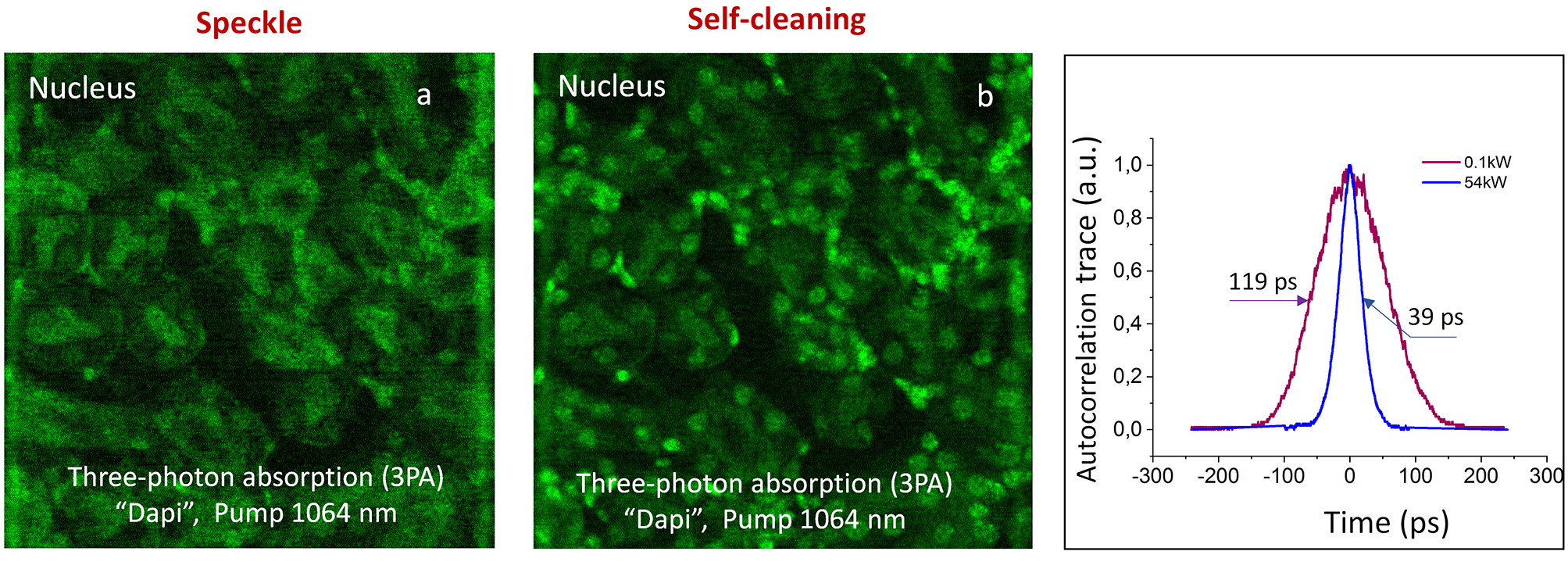


**c**

**Figure SM4.** Illustration of pulse peak power increase after self-cleaning process at the pump wavelength. (**a**) Three-photon fluorescence image of mouse kidney cells labelled with DAPI, recorded by using a speckled beam at the GRIN MMF output. (**b**) The same zone, by using a self-cleaned beam at the GRIN MMF output. (**c**) Pulse autocorrelation traces taken at the fiber output for either a low peak power (0.1kW), or for spatially self-cleaned beam (54 kW). Dwell time: 5µs, repetition rate: 200 kHz, average power on the sample: 16 mW.

However, because of the remaining energy in the speckled background, and by limiting the calculation of the peak power to the energy localized in the beam center which is mainly populated by the fundamental mode, we estimated a net peak power improvement factor of about 2. Indeed, as it is shown in **Fig. SM4**, the interplay between beam brightness and peak power increase allowed us to significantly improve the performance of nonlinear imaging. We recorded an image of mouse kidney by imaging nucleus of cells, labelled by DAPI. As seen in the **Fig. SM4a**, for 16 mW of average pump power, no identification of nucleus could be obtained with a speckled beam. However, with the same average power level, the nucleus became clearly visible on the image, when spatiotemporal beam reshaping is exploited (**Fig. SM4b**).

**References**

1. Krupa, K. et al. Spatial beam self-cleaning in multimode fibers. *Nat. Photonics*, **11**, 237–241 (2017).
2. Krupa, K. et al. Multimode nonlinear fiber optics, a spatiotemporal avenue. *APL Photonics*. **4**, 110901 (2019).
3. Krupa, K. et al. Spatiotemporal light-beam compression from nonlinear mode coupling. *Phys. Rev. A* **97**, 043836 (2018).
